# An Improved Algorithm for the Piecewise-Smooth Mumford and Shah Model in Image Segmentation

**DOI:** 10.1155/BSB/2006/84397

**Published:** 2006-04-04

**Authors:** Yingjie Zhang

**Affiliations:** 1 School of Mechanical Engineering, Xi'an Jiaotong University, Xi'an Shaanxi 710049, China

## Abstract

An improved algorithm for the piecewise-smooth Mumford and Shah functional is presented. Compared to the previous work of Chan and Vese, and Choi et al., extensions of the key functions  are replaced by updating the level set function based on an artificial image that is composed of the diffused image and the original image. The low convergence problem of the classical algorithm is efficiently solved in the proposed approach. The resulting algorithm has also been demonstrated by several cases.

## 

[1,2,3,4,5,6,7,8,9,10,11]
